# Multiple tissue-specific epigenetic alterations regulate persistent gene expression changes following developmental DES exposure in mouse reproductive tissues

**DOI:** 10.1080/15592294.2022.2139986

**Published:** 2022-11-03

**Authors:** Tanner B. Jefferson, Tianyuan Wang, Wendy N. Jefferson, Yin Li, Katherine J. Hamilton, Paul A. Wade, Carmen J. Williams, Kenneth S. Korach

**Affiliations:** a Reproductive and Developmental Biology Laboratory, National Institute of Environmental Health Sciences, National Institutes of Health, Durham, NC, 27709, USA; b Integrative Bioinformatics, National Institute of Environmental Health Sciences, National Institutes of Health, Durham, NC, 27709, USA; c Epigenetics and Stem Cell Biology Laboratory, National Institute of Environmental Health Sciences, National Institutes of Health, Durham, NC, 27709, USA

**Keywords:** Estrogen receptor, transcriptome, histone modification, neonatal DES exposure, mouse seminal vesicles

## Abstract

Clinically, developmental exposure to the endocrine disrupting chemical, diethylstilboestrol (DES), results in long-term male and female infertility. Experimentally, developmental exposure to DES results in abnormal reproductive tract phenotypes in male and female mice. Previously, we reported that neonatal DES exposure causes ERα-mediated aberrations in the transcriptome and in DNA methylation in seminal vesicles (SVs) of adult mice. However, only a subset of DES-altered genes could be explained by changes in DNA methylation. We hypothesized that alterations in histone modification may also contribute to the altered transcriptome during SV development. To test this idea, we performed a series of genome-wide analyses of mouse SVs at pubertal and adult developmental stages in control and DES-exposed wild-type and ERα knockout mice. Neonatal DES exposure altered ERα-mediated mRNA and lncRNA expression in adult SV, including genes encoding chromatin-modifying proteins that can impact histone H3K27ac modification. H3K27ac patterns, particularly at enhancers, and DNA methylation were reprogrammed over time during normal SV development and after DES exposure. Some of these reprogramming changes were ERα-dependent, but others were ERα-independent. A substantial number of DES-altered genes had differential H3K27ac peaks at nearby enhancers. Comparison of gene expression changes, H3K27ac marks and DNA methylation marks between adult SV and adult uterine tissue from ovariectomized mice neonatally exposed to DES revealed that most of the epigenetic changes and altered genes were distinct in the two tissues. These findings indicate that the effects of developmental DES exposure cause reprogramming of reproductive tract tissue differentiation through multiple epigenetic mechanisms.

## Introduction

Early exposure to endocrine disrupting chemicals (EDCs) causes reproductive dysfunction in adulthood [1,2]. This dysfunction manifests as reproductive diseases and abnormalities in adults in both human and animal studies [3]. Diethylstilboestrol (DES) is a potent synthetic oestrogen and a known EDC [4–6]. Based on human clinical abnormalities caused by prenatal DES exposure, a mouse model of DES exposure was developed to study the full effects of this chemical on the developing reproductive system [7–9]. Male and female reproductive organs have a functional requirement for oestrogen receptors (ERs) [2]. There are two ERs, ERα (encoded by *Esr1*) and ERβ (encoded by *Esr2*), that are members of a large superfamily of nuclear receptors that modulate transcription following ligand binding [10,11]. EDCs utilize the same ERs as natural occurring endogenous oestrogens such as oestradiol. Exposure to EDCs during key developmental stages can interfere with many normal ER-dependent processes [12]. EDCs can interfere with oestradiol’s ability to create functional reproductive organs during development and are specifically associated with reproductive dysfunction in adulthood [11].

Estrogen plays several important roles in adult male reproductive tract function. Estrogen has direct effects on Leydig cells and the efferent duct epithelium as well as potential effects on germ cells [13]. One of oestrogen’s primary functions in the male reproductive tract is fluid reabsorption from the efferent ductules [13]. Oestrogen also influences seminal vesicle and prostate weight directly, suggesting it has important functions in these tissues [14].

To understand the role of ERs in mediating EDC effects on the development of the reproductive tract of male mice, global ERα-knockout (αERKO) mouse models have been combined with a well characterized neonatal DES exposure paradigm (2 µg daily on postnatal days 1–5) [8,15]. Female CD-1 mice exposed neonatally to DES at this dose exhibit complete infertility and a high incidence of uterine adenocarcinoma by 12 months of age; αERKO mice are refractory to most of these effects [8,15]. Of note, αERKO female mice are on a C57BL/6 background and wild-type C57BL/6 mice do not develop uterine adenocarcinoma, even though they exhibit atypical hyperplasia and other phenotypes observed in CD-1 mice. For this reason, most studies of epigenetic responses to DES exposure are not performed in C57BL/6 mice. Male rodents exposed perinatally to DES exhibit disrupted spermatogenesis, testicular tumours and altered reproductive tract morphology with severe reductions in prostate and seminal vesicle (SV) weight [16–19]. Similar to humans, the prostate and SV are required for fertility in mice, reductions in their size and function most likely contribute to the infertility observed following DES exposure to males [20].

The impact of developmental DES exposure on the male reproductive tract is mediated by ERα as αERKO mice exhibit normal reproductive tract weight [15,19,21–23]. Of note, ERβ is expressed at low levels in some male reproductive tissues; however, global ERβ-knockout mice exposed neonatally to DES exhibit a wild-type (WT) phenotype, suggesting DES acts predominantly through ERα in male reproductive tract tissues during neonatal DES exposure [19,22,24].

The DES-induced phenotype is characterized by resistance to androgen stimulation most likely due to lower levels of androgen receptor in both prostate and SV despite normal circulating levels of testosterone [19,22,25]. Functional androgen resistance is also observed in these tissues with reduced levels of downstream androgen regulated genes such as *Svs4* [22]. In addition to androgen resistance, DES exposed male reproductive tract tissues also exhibit a molecular feminization phenotype with higher expression of ERα as well as aberrant expression of oestrogen regulated genes such as *Ltf* [22]. These data taken together show altered differentiation of the male reproductive tract towards a more female fate, but the mechanism underlying this change is largely unknown.

Regulation of epigenetic marks is a common mechanism for modulating nuclear receptor function. Consequently, modification of the epigenetic landscape can cause physiological dysfunction [26]. Alteration in DNA methylation is one example of epigenome changes that can lead to changes in gene expression [27]. Neonatal DES exposure alters ERα-mediated *Ltf* and *Svs4* gene expression in mouse SVs and those changes are associated with altered DNA methylation patterns [21]. On a global scale, DNA methylation patterns are altered in the SV following neonatal DES exposure and expression levels of about 60% of DES-altered genes are attributed to DNA methylation status near these genes [1]. Some of the DES induced alterations are dependent on ERα but others are unique to the loss of ERα suggesting a role for ERα in DNA methylation status.

The remaining 40% of DES-altered gene expression is not accounted for by DNA methylation changes and suggests there are other modifications to the epigenome to explain these changes. Histone modifications are another major source of epigenetic control of gene expression changes [27,28]. These modifications predominantly consist of posttranslational modifications of the tails of the core histones 2 (H2), 3 (H3), and 4 (H4) [28]. Acetylation of core histones is typically associated with gene activation and methylation of core histones is typically associated with gene repression; however, this is not always the case. There is some relationship between DNA methylation and histone marks; for example, DNA methylation is influenced by H3K9me3 association [29]. In support of altered histone modifications, a recent study showed excessive accumulation of the histone modification, histone H3K27ac at enhancers near DES-altered genes in the uterus of female mice during the time of exposure [30]. In addition, these changes are largely dependent on ERα because lack of ERα prevented this effect. The impact of neonatal DES exposure on this epigenetic modification in the female suggests similar effects may be found in male reproductive tract tissues following neonatal exposure to DES.

The current study examines neonatal DES induced alterations in H3K27ac association in the pubertal and adult SV. The dose of DES used in this study results in robust changes in SV phenotype with a very high penetrance (all mice exhibit the phenotype) [19]. We have previously examined RNA expression and DNA methylation patterns in the adult SV from similarly exposed mice, allowing us to overlap the data obtained in this study with the current study [1]. We also determine the dependence of these changes on ERα and their relationship to gene expression changes. Lastly, we explore tissue specificity of DES induced epigenetic changes by comparing data generated in DES exposed adult SV with DES exposed ovariectomized adult female uterus (UT). Previous studies show a robust change in the uterine epigenome following neonatal DES exposure in CD-1 female mice and suggest this tissue as a good comparison of tissue specificity against the SV; CD-1 mice were used due to limited phenotype changes in C57BL/6 mice [15,30].

## Methods

### Animal and neonatal treatments

All animal studies were conducted in accordance with the NIH Guide for the Care and Use of Laboratory Animals. The procedures were described previously [21,22]. Briefly, for the wild type (WT) mice, pregnant C57Bl/6 females were obtained from Charles River Laboratories. ERα null mice (αERKO) were generated by breeding C57Bl/6 heterozygous (ERα±) animals [31]. For neonatal treatment, as shown in figure S1, all male pups were treated by subcutaneous injection with DES (Sigma-Aldrich), dissolved in corn oil at 2 µg/pup/day on days 1–5 (DES group) or an equal volume of corn oil (vehicle group). This dose regime of DES causes robust changes in male reproductive tract tissues and has been used in a previous study examining DNA methylation that will be compared to our current study [1]. Mice were weaned at 21 days of age, housed 2–4 per cage, and αERKO mice were genotyped by Transnetyx from ear biopsy. The seminal vesicle (SV) tissues were dissected away from the anterior prostate at week 5, and 10 from vehicle (veh) or DES-treated groups and snap frozen for future use [21,22,32]. For uterine tissue, CD-1 female pups were treated, weaned and housed as described for male C57BL/6 mice above. Uterine tissues were collected at week 10, 14 days following ovariectomy from WT CD-1 mice after DES neonatal exposure as described previously [1].

### RNA extraction and qPCR

Total RNA samples were extracted from frozen SV (week 5 and week 10) or uterine (week 10) tissues of four individual mice for WT-veh, αERKO-veh, or αERKO-DES group samples, and three pools [5–8] for WT-DES group samples using trizol and RNeasy Mini Kit (Qiagen). For qPCR, first-strand cDNA synthesis was performed using Superscript reverse transcriptase (Invitrogen). The mRNA levels of genes were measured using SYBR green assays (Applied Biosystems). Cycle threshold (Ct) values were obtained using the ABI PRISM 7900 Sequence Detection System and analysis software. The experiments were repeated three times and results are presented as fold increase calculated relative to the vehicle of WT SV ± SE.

### RNA-seq, mapping, and analysis

RNA-Seq was performed at NIEHS Epigenetic Core facility for WT or αERKO SVs (week 5 and week 10) or WT UT (week 10), four samples per each treatment group. The RNA libraries were sequenced with a HiSeq 2000 system [Illumina]. The raw RNA-seq reads (126nt, paired-end) were initially processed by filtering with average quality scores greater than 20. The reads passing the initial processing were aligned to the mouse reference genome (mm10; Genome Reference Consortium Mouse Build 38 from December 2011) using TopHat version 2.0.4 [21] and assembled using Cufflinks version 2.0.2 [33]. Mapped reads were converted to coverage and displayed on the UCSC genome browser as custom tracks. Expression values were displayed as FPKM (fragments per kilobase of exon per million fragments) values. For mRNAs data, differential expression genes (DEGs) were calculated using Cuffdiff [33]. Transcripts with q‐value ≤ 0.05, at least a 1.5-fold difference, and with average FPKM > 1 in at least one group were defined as DEGs. Hierarchical clustering of DEGs was generated by the Genomics Suite of Partek software package version 6.6. Functional analysis of DEGs was performed using the Ingenuity Pathway Analysis (IPA, www.ingenuity.com) based on the content of 2015–03-22. For lncRNA data, the analysis method is the same as mRNA analysis described above, except using the GENCORE VM21 lnc RNA gene annotation (gencode.vM21.long_noncoding_RNAs.gtf) was downloaded from the website, https://www.gencodegenes.org/mouse/release_M21.html. For a given biological category in IPA, Fisher’s exact test was used to measure the probability (p-value) that the category was randomly associated. The categories with p-values less than 0.05 were defined as significantly enriched. Base Space Correlation Engine was used to identify curated data sets in their knockout atlas that have the highest overlap with DEG list (Illumina).

### ELISA assay

A standard curve was initially prepared by using a serial dilution of recombinant histones provided by the manufacturer (Active Motif). 1 µg of acid extracted histones were diluted with dilution buffer and incubated at RT on an orbital shaker at low speed for 1 h. Wells were then washed 3 times with wash buffer and primary antibody (Active Motif H3K27ac cat# 104602 1:2000) was diluted in dilution buffer. The diluted primary antibody was added to each of the wells containing samples. The plate was incubated at RT on an orbital shaker at low speed for 1 h and washed 3 times with wash buffer. Secondary antibody (Active Motif HRP anti-mouse IgG1 cat# 104603 1:2000) was diluted in dilution buffer and added to the wells containing samples. The plate was incubated at RT without agitation and washed 3 times with wash buffer. The plate was dried by blotting onto a paper towel and air dried for an additional 10 minutes. RT developing solution was added to each well containing a sample and was incubated at RT wrapped in foil for ~5 mins (until the colour became a medium to dark blue). Stop solution was added and the absorbance was read on a spectrophotometer immediately at 450 and 655 nm.

### Histone extraction and immunoblot analysis

Cytoplasmic and nuclear fractions of samples were extracted from frozen tissues of each group using Nuclear and Cytoplasmic Extraction Reagents (Thermo Fisher) as per the manufacturers protocol. The pellet remaining after the nuclear and cytoplasmic extractions was soaked in 0.4 N H_2_SO_4_ on ice for 1 h. Acetone was added to the histones precipitated out overnight at −20°C. Protein concentrations were quantified using protein assay on a Qubit fluorometer (Invitrogen). 2 ug of histones were loaded into a premade 4–20% gradient acrylamide gel (Bio-Rad) and ran for 40 minutes at 200 V. Gels were transferred onto nitrocellulose membranes using i-Blot 7-min transfer system (Invitrogen). Blots were blocked using 5% milk in TBS-T for 1 h at room temperature. After blocking, the blots were incubated overnight with primary antibody (Millipore Total H3 cat# 92590 1:1000; Active motif H3K27ac cat#39133 1:1000; Active Motif H3K27me3 cat# 39155 1:1000) diluted in 5% milk/TBS-T at 4°C. The blots were washed using TBS-T and then incubated with IRdye 800cw goat anti-mouse fluorescent antibody (Li-Cor) in 5% milk/TBS-T for 1 h at room temperature. The blots were imaged using the Li-Cor Fc system.

### H3K27ac ChIP-seq, mapping, and analysis

The SV samples were collected from the 5 or 10-weeks-old either WT or αERKO mice and the UT samples were collected from the 10-weeks-old WT mice. Frozen tissues (pooled from 5 to 15 mice) were sent to ACTIVE MOTIF for the assay. The raw ChIP-seq reads (706 nt, single-end) were filtered with average quality scores greater than 20. Then the reads were mapped to the mouse reference genome (mm10; Genome Reference Consortium Mouse Build 38 from December 2011) using Bowtie version 1.1.2 (Langmead et al., 2009) with unique mapping and allowing up to 2 mismatches for each read. The duplicated reads with the same sequence were removed. The normalized read coverage was displayed on UCSC genome browser as custom tracks. Heatmaps of histone mark signal were generated by the Genomics Suite of Partek software package version 6.6 with 50bp bin (Partek Inc., St. Louis, MO, USA). The histone mark signal distribution around promoter region (TSS ± 5Kb) was represented as the average reads per million uniquely mapped reads (RPM). Software MEDIP was used to identify differential regions of histone mark between any pair of ChIP-seq samples. Each region was defined as the genomic interval with at least 2-fold differences of read count and p‐value ≤ 0.01. Each differential regions were mapped to nearby gene within 100Kb of transcription start site (TSS) using software HOMER’s ‘annotatePeaks.pl’ function (Heinz et al., 2010). All the histone mark differential regions were assigned to genomic regions using web application PAVIS (Huang et al., 2013). The motif analysis of differential histone mark regions was performed using software HOMER’s ‘findMotifsGenome.pl’ function with default setting (Heinz et al., 2010).

### DNA extraction, DNA methylation (MBD) ChIP-seq, mapping, and analysis in the uterine tissue

It was used the same protocol for the SV tissues as described previously [1]. Genomic DNA samples were extracted from a pool of 5–18 frozen uterine tissues (week 10) of for each group using a Tissue Blood Kit (Qiagen, Valencia, CA) according to the manufacturer’s protocol. Pooled genomic DNA samples were sonicated with Biorupter (Diogenode), and methylated DNAs were captured with his-tagged recombinant MBD2b along with its binding partner MBD3L1 using MethyColletor Ultra kit (cat# 55005, ACTIVE MOTIF). The MBD ChIP-seq libraries were prepared using the TruSeq kit (Illumina, San Diego, CA) and the sequencing was conducted in the Epigenetic Core at NIEHS/NIH with a MiSeq system (Illumina). The raw MBD-Seq reads (51nt, paired-end) were processed by filtering for average quality scores greater than 20. The reads passing the initial processing were aligned to the mouse reference genome (mm10; Genome Reference Consortium Mouse Build 38 from December 2011) using Bowtie version 1.1.2 [4] with unique mapping and up to 2 mismatches for each read. Duplicated reads were discarded. To achieve same sequencing depth, 5 million uniquely mapped paired-end reads from each sample were randomly selected for future analyses.

### Data deposition

RNA-seq for week 10 WT and αERKO SVs and WT UTs, and MBD ChIP-seq for weeks 5 and 10 WT and αERKO SVs were described previously [1]. RNA-seq for SV week 5 in WT and αERKO (veh and DES), H3K27ac ChIP-seq for SV week 5 and week 10 in WT and αERKO (veh and DES), and UT week 10 in WT (veh and DES), MBD ChIP-seq for UT week 10 in WT (veh and DES) were deposited in GEO with accession number GSE152526.

### Statistical analysis

One-way ANOVA with Multiple Comparison test were performed using GraphPad Prism version 8.2.1 for Figures 2e, 2f.

## Results

### Impact of neonatal DES exposure on the developing seminal vesicle transcriptome

**Figure 1.**
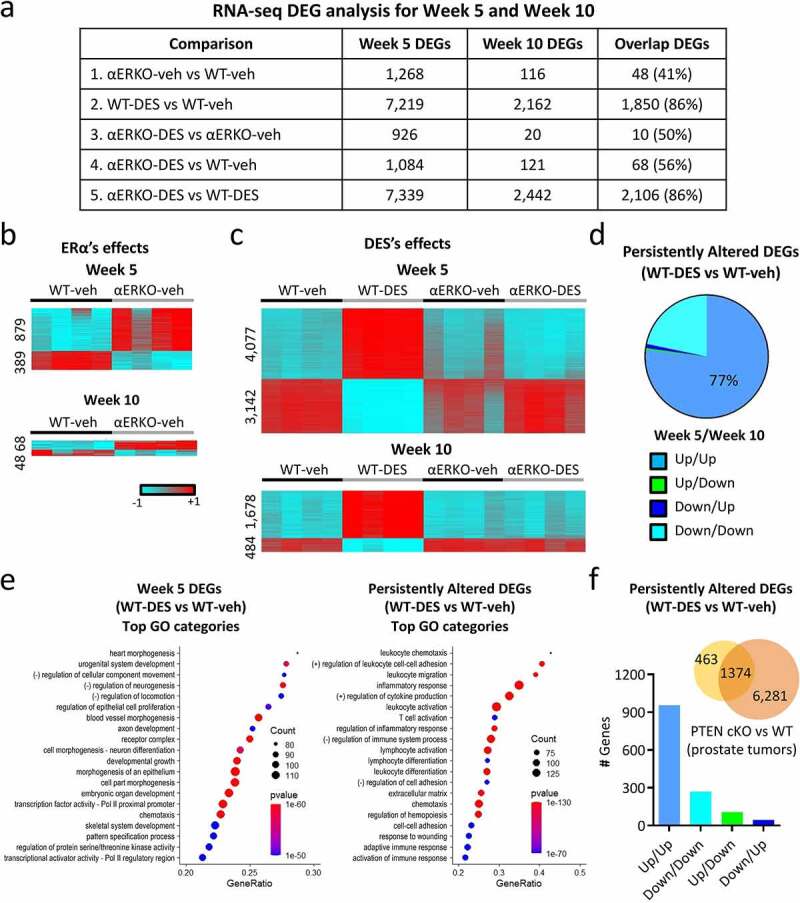
mRNA transcriptome changes in WT and αERKO mouse SVs during normal development and after neonatal DES exposure. (a) RNA-seq DEG analysis for week 5 and week 10 mouse SVs as well as the overlap of both groups. (b) Heat maps displaying DEGs (αERKO-veh versus WT-veh) at week 5 and week 10. Heat map is plotted using z-scores. (c) Heat maps displaying DEGs (WT-DES versus WT-veh) at week 5 and week 10. (d) Persistently altered DEGs in WT-DES vs. WT-veh (overlap of week 5 and week 10 DEGs). DEGs are split into the four indicated categories. (e) Go categories analysis for week 5 DEGs (WT-DES versus WT-veh) and persistently altered DEGs. (f) Overlap of persistently altered DEGs and DEGs from *Pten* conditional prostate epithelial KO (prostate tumours) vs WT (normal prostate). Graph plotted of correlation of gene expression change direction from both datasets.

To further investigate the range of developmental effects due to DES exposure, we performed RNA-Seq analysis on 5-week-old mice (puberty) after neonatal DES exposure and compared this data to our previously acquired 10-week SV RNA-Seq data (Figure 1).

Differentially expressed genes (DEGs) were analysed by examining fold changes with ±1.5-fold cut-off; a complete list of the comparisons is summarized in Figure 1a (DEG numbers) and table S1 (DEG lists). In all comparisons, there were more DEGs at week 5 when compared to week 10, suggesting an influence of development on gene expression changes. To further investigate these developmental changes, we also performed an overlap of DEGs between week 5 and week 10 of each of the comparisons (Figure 1a).

In control WT-veh SVs, a large developmental change occurred in the mRNA transcriptome between puberty and adulthood, with 6,882 differentially expressed genes (DEGs) (table S2). A comparison of SVs from vehicle-exposed WT and αERKO mice at week 5 revealed 1,268 DEGs (879 up- and 389 down-regulated) (Figures 1a, 1b). However, only a minimal difference was seen in adult SV gene expression between WT and αERKO mice, with only 116 DEGs (68 up- and 48 down-regulated) at week 10 and only 48 of those overlapping with week 5 DEGs. These findings indicated that pubertal changes in SV gene expression depend in part on endogenous oestrogen signalling through ERα, whereas ERα signalling minimally impacts the adult SVs.

There was a robust change in SV gene expression following neonatal DES exposure with 7,219 DEGs (4,077 up- and 3,142 down-regulated) relative to WT controls at 5 weeks of age (Figure 1a). At week 10, the difference in the mRNA transcriptomes was far less than week 5, with only 2,162 mRNA DEGs (1,678 up- and 484 down-regulated), most of which were upregulated in the DES group (Figures 1a, 1c). There was also a substantial overlap (86%) of the week 10 DES-induced gene expression changes with week 5 demonstrating many genes are persistently altered following developmental exposure to DES. Additionally, most of these genes were persistently altered in the same direction at both week 5 and week 10 with 77% of them increased at both time points (Figure 1d). These data suggest a substantial persistent impact of neonatal DES exposure on the adult SV.

To determine the impact of ERα on DES-induced DEGs, we performed three comparisons (Figure 1a). The first was a comparison of αERKO-DES to αERKO-veh (Figure 1a, comparison 3) where ERα independent effects of DES would be observed. There was minimal change in gene expression at week 5 (926 DEGs) and almost no change at week 10 (20 DEGs). Another comparison to examine ERα independent effects of DES was between αERKO-DES and WT-veh (Figure 1a, comparison 4). Again, there were very few gene expression changes with 1,084 DEGs on week 5 but only 121 DEGs on week 10 (56% overlap with week 5) suggesting very few ERα independent DES-induced changes in SV gene expression. The third comparison was between αERKO-DES and WT-DES (Figure 1a, comparison 5) where the most direct ERα-dependent DES changes were observed. As expected, many gene expression changes were dependent on ERα with 7,339 DEGs on week 5 and 2,442 DEGs on week 10. There was also a very high overlap of the week 10 DEGs with the week 5 DEGs (86%). These data taken together showed the impact of neonatal DES exposure on the adult SV was predominantly mediated through ERα.

Next, we determined if there were any changes in the expression of long non-coding RNAs (lncRNAs). The four groups of comparisons from differentially expressed regions (DE lncRNAs) of the lncRNA analysis are summarized in Table S3. We found that 1,188 lncRNA regions were affected at the 5-week time point due to DES treatment and only 607 at 10 weeks (table S3, comparison 1, WT-DES vs WT-veh). However, αERKO mice were impacted much less by DES treatment with 169 and 34 altered lncRNAs at week 5 and 10, respectively (table S3, comparison 2: αERKO-DES vs αERKO-veh). Similar to coding RNA, there were a number of lncRNAs that were altered in the absence of ERα without DES exposure (355 at week 5; table S3, comparison 3: WT-veh vs αERKO-veh) but again this effect was largely eliminated by adulthood (50 at week 10; table S3, comparison 3: WT-veh vs αERKO-veh) supporting the natural transient ERα-dependent RNA expression changes in the SV. A comparison of WT-DES with αERKO-DES at week 5 showed 1,191 DEGs (table S3, comparison 4: WT-DES vs αERKO-DES) which was similar to the number found between WT-DES and WT-veh (1,188 DEGs). These results indicated that DES has a large persistent impact on the whole SV transcriptome, not only on coding RNAs but also on lnc RNAs that appeared mediated through ERα during development following neonatal DES exposure.

To determine if specific SV functions were disrupted by DES exposure over time, Base Space Correlation Engine Pathway Analysis was performed on the 5,369 week 5 specific transient DES altered DEGs and the 1,850 persistent DES altered DEGs (Figure 1e). The top 20 most significant GO categories in the week 5 transiently altered DEGs were cell growth, morphogenesis and proliferation while the persistently altered gene categories were inflammatory and immune response along with extracellular matrix. These data suggested DES interferes with developmental processes during puberty but alters immune function and inflammatory response in a persistent manner. An additional analysis comparing the persistently altered DEGs to the Base Space Correlation Engine knockout (KO) atlas revealed *Pten* as one of the most highly significant perturbed genes. One of the top examples from the highly overlapped *Pten* knockout datasets was from prostate tumour tissue of *Pten* KO mice compared to normal wild-type prostate tissue (Figure 1f). Almost all of the overlapping persistent DES-altered genes [1,374] were altered in the same direction suggesting our model is highly correlative to this model.

### Neonatal DES exposure acts through ERα to persistently change chromatin modifying proteins in mouse seminal vesicles

**Figure 2.**
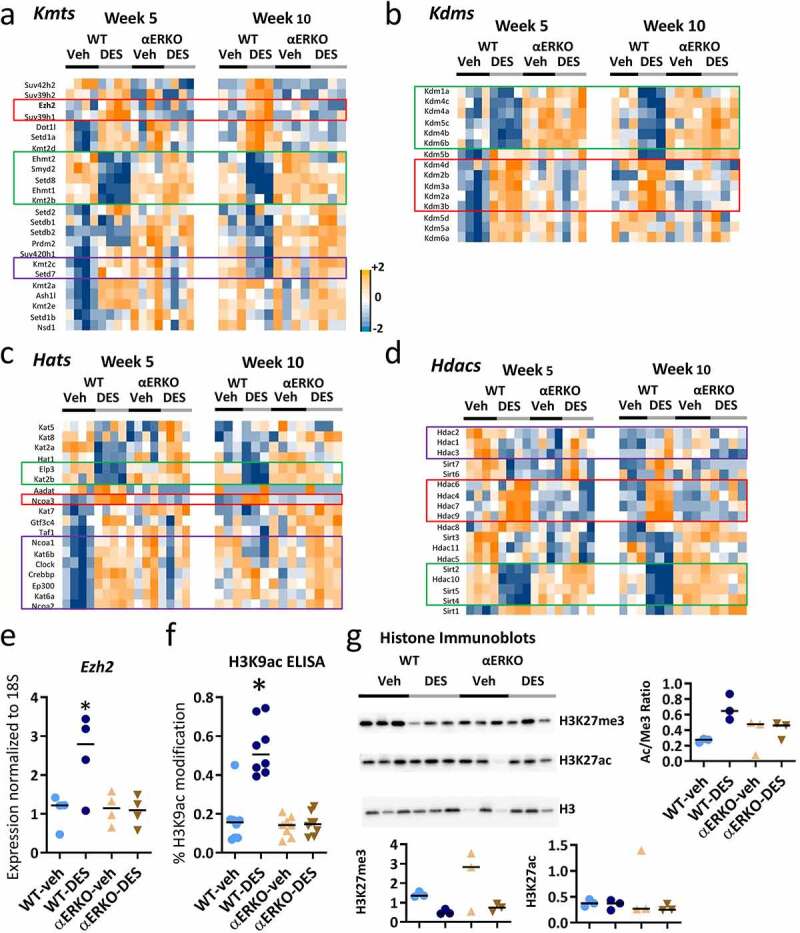
Expression of histone modifying enzymes and activity is altered in SVs following neonatal DES exposure. Impact of DES treatment in WT and αERKO mice on genes associated with the following types of histones modifying genes (a-d). (a) Lysine methyltransferases (*Kmts*). (b) Lysine demethylases (*Kdms*). (c) Lysine acetyltransferases (*Kats*). (d) Lysine deacetylases (*Hdacs*). Heat maps of gene expression (*log2* normalized FPKM) were plotted for each indicated category at week 5 and week 10. (e) Real time RT-PCR of *Ezh2* in SV at 10 weeks in WT-veh, WT-DES, αERKO-veh and αERKO-DES (n = 4 per group). Numbers shown are *Ezh2* expression normalized to *18S* and then fold change calculated using the average of the WT-veh group set to 1. *p-value<0.01 using Mann Whitney. (f) ELISA assay analysis on H3K9ac. (g) Histone H3K27me3, H3K27ac and total H3 immunoblots for each indicated category at week 10 (n = 3 individual mice per group). H3K27me3 and H3K27ac were normalized to total H3, and values plotted in graphs.

Persistent changes in gene expression can be driven by alterations in epigenetic marks that are associated with changes in levels of chromatin modifying proteins [34–39]. To determine the types of epigenetic changes in SV tissue that might occur in response to developmental DES exposure, we examined our RNA-seq datasets for the expression of genes encoding regulators of histone methylation and acetylation [40]. Most lysine methyltransferases (*Kmts*) analysed had alterations due to DES at both 5 and 10 weeks and many of these changes were dependent on ERα (Figure 2a). For example, several H3K4 methyltransferases, including *Ehmt2, Smyd2, Setd, Ehmt1*, and *Kmt2b*, were all down regulated by DES at both 5 and 10 weeks and the lack of ERα prevented this down regulation. The converse was true for two H3K27 methyltransferases, *Ezh2* and *Suv39h1*, which were upregulated in response to DES. A few of the *Kmts* exhibited up regulation at week 5 but down regulation at week 10, including *Kmt2c* and *Setd7*. Some genes, such as the H3K9 methyltransferases *Setdb1* and *Setdb2*, were altered in the absence of ERα, independent of DES exposure.

Most lysine demethylases (*Kdms*) fell into two categories: up- or down-regulated and protected by lack of ERα at both 5 and 10 weeks (Figure 2b). The H3K9 demethylases *Kdm1a, Kdm4c, Kdm4a*, and *Kdm4b* were persistently downregulated due to DES treatment. Another group of *Kdms* that demethylate H3K9 (*Kdm3a, Kdm3b*, and *Kdm4d*) or H3K36 (*Kdm2a* and *Kdm2*) were persistently upregulated following DES exposure and protected by lack of ERα. None of the selected *Kdms* appeared to have been impacted by loss of ERα alone.

Many histone acetyltransferases (*Hats*) had a robust increase in gene expression at week 5 following neonatal DES exposure. These include *Hats* that are involved in nuclear receptor-dependent transcription such as *Ncoa1, Crebbp, Ep300*, and *Ncoa2* [26,28] (Figure 2c). *Hats* in this group were also upregulated in αERKO compared to WT mice, independent of DES treatment. The expression pattern for these *Hats* was quite different at week 10, with some exhibiting ERα-dependent decreases and others exhibiting ERα-independent increases, indicating that their regulation diverged over time during this developmental period. *Ncoa3* was increased at weeks 5 and 10 and was ERα-dependent, whereas two other *Hat* genes, *Elp3* and *Kat2b*, were robustly decreased at both time points in an ERα-dependent manner.

Like the other histone modifiers, histone deacetylases (*Hdacs*) had a variety of gene expression patterns (Figure 2d). One group of Hdacs (*Hdac4, Hdac6, Hdac7* and *Hdac9)* were robustly upregulated and another group (*Sirt2, Sirt4, Sirt5* and *Hdac10*) were robustly downregulated at both weeks 5 and 10 in an ERα-dependent manner. *Hdac2, Hdac1*, and *Hdac3* were downregulated by DES at week 5 but upregulated or unchanged by DES at week 10, indicating a developmental change in their regulation.

To validate the RNA-seq data, we checked *Ezh2* expression by qPCR. There was a significant increase in *Ezh2* mRNA similar to the RNA-seq data that is not observed in either αERKO group (Figure 2e). To determine if there were any functional consequences of the changes in Hat and Hdac gene expression following neonatal DES exposure, we examined the global levels of several modified histones. An ELISA assay for H3K9ac showed a significant increase in H3K9ac in adult DES-exposed WT mice and that increase was dependent on the presence of ERα (Figure 2f). A histone H3K27me3 immunoblot normalized to total histone H3 showed a significant reduction in global levels in WT-DES SV compared to WT-veh SV or αERKO-veh (Figure 2g). There was also an apparent reduction in levels of histone H3K27me3 in αERKO-DES compared to αERKO-veh but this difference was not significant most likely due to the variability observed in the αERKO-veh group. The DES decreased H3K27me3 was in conflict with significantly increased *Ezh2*, the *Kmt* responsible for tri-methylation of H3K27 as well as the decreased *Kdms, Kdm1a* and *Kdm6b*, responsible for demethlating H3K27; however, increased *Kdm6a*, another *Kdm* responsible for demethylating H3K27 is in agreement with this finding [41–43]. A histone H3K27ac immunoblot showed similar global levels of this modified histone across all four groups (Figure 2g; left panel). The ratio of H3K27ac to H3K27me3 was increased in WT-DES compared to WT-veh due to the reduced levels of histone H3K27me3. In addition, this increased ratio in the WT-DES was approaching when compared to either of the αERKO groups demonstrating the dependence on ERα for this effect. These data indicated that there are persistent alterations in SV histone acetylation and methylation following a brief developmental exposure to DES although the gene expression of enzymes responsible for these modifications does not always correspond to the global levels observed.

### Neonatal DES exposure alters histone H3K27 acetylation patterns during seminal vesicle development

Alterations in histone modifying enzymes as well as impact on global levels of some modified histones following neonatal DES exposure suggested that the epigenetic landscape of modified histones was a likely mediator of persistent changes in SV gene expression. In a previous study, histone H3K27ac ChIP-seq in neonatal uteri following DES exposure showed robust changes in association at specific genomic locations when compared to controls despite similar levels of H3K27ac across treatment groups [30,34]. Additionally, H3K27ac is commonly associated with active transcription and also can identify active enhancers [29]. To determine if there are alterations in the SV epigenome and if these changes are ERα dependent, we performed histone H3K27ac ChIP-seq on week 5 and week 10 WT-veh, WT-DES, αERKO-veh and αERKO-DES SV.

**Figure 3.**
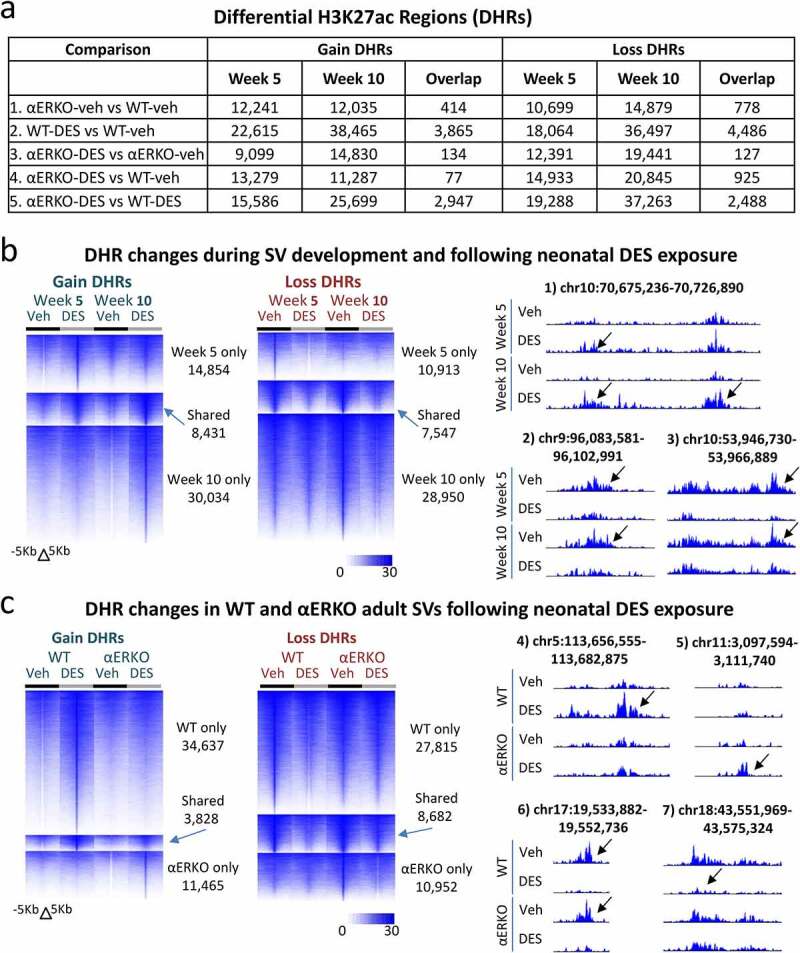
Histone H3K27ac changes in WT and αERKO mouse SVs during normal development and after DES exposure. (a) H3K27ac ChIP-seq DHRs (gain and loss) analysis in SVs at week 5 and week 10 as well as the overlap of these two time points. The comparison being made is indicated on each row of the table. (b) Heat maps displaying DHRs separated by gain and loss (WT-DES versus WT-veh) during SV development and following neonatal DES exposure in WT mice (left). UCSC browser tracks displaying example regions with DHRs (right). Black arrows denote region that is different due to treatment or genotype. Chromosome locations are indicated above each track. (c) Heat maps displaying DHRs separated by gain and loss (WT-DES versus WT-veh and ERKO-DES versus αERKO-veh) in WT and αERKO adult SVs following neonatal exposure to DES exposure (left). UCSC browser tracks displaying example regions with DHRs (right). Black arrows denote region that is different due to treatment or genotype. Chromosome locations are indicated above each track.

Similar to normal developmental gene expression changes, there were a large number of differential H3K27ac regions (DHRs) between week 5 WT-veh and week 10 WT-veh. There were 25,260 DHRs gained (week 10 WT-veh versus week 5 WT-veh) showing a robust acquisition of H3K27ac region in adulthood compared to the pubertal period; in contrast there were only 9,904 DHRs lost (week 10 WT-veh versus week 5 WT-veh). The number of DHRs gained or lost when comparing the different treatment/genotype groups are summarized in Figure 3a.

The largest number of DHRs were found in the WT-DES versus WT-veh group showing that neonatal DES exposure exhibited a larger impact on the SV epigenome than that occurring from lack of ERα (Figure 3a). The genomic locations of DHRs in all comparisons were largely in introns and intergenic spaces for all groups, indicating that DHRs were located mainly in enhancer regions regardless of treatment or genotype (figure S2).

To determine the impact of ERα on the epigenome during development of the SV from puberty to adulthood, we compared DHRs between αERKO-veh and WT-veh. The lack of ERα in vehicle treated mice resulted in ~12,000 DHRs equally split between gain and loss despite little change in gene expression and no apparent DEG changes in phenotype between αERKO-DES and αERKO-veh (Figures 1a, 3a). Overlap of the DHRs between these two time points again showed almost no overlap (414 gain and 778 loss) demonstrating the transient nature of DHRs between puberty and adulthood. This group of DHRs confirmed the lack of ERα over the course of SV development from puberty to adulthood did not impact the epigenome in a persistent manner.

A large number of DHRs (gain and loss) were observed in WT DES-exposed mice when compared to WT controls at both week 5 and 10 and the numbers gained and lost for each time point were similar (Figure 3a). Interestingly, there were approximately twice as many DHRs (both gain and loss) at week 10 compared to week 5 suggesting further perturbation over time. An overlap of the DHRs between the two time points showed ~21% of all of the week 5 DHRs were found in common at week 10 demonstrating persistence of the impact of neonatal DES exposure on the H3K27ac pattern across the developmental time period (Figure 3a). Heat maps of the DHRs for this comparison showed robust changes in H3K27ac ChIP-seq signal between WT-DES and WT-veh at both 5 weeks and 10 weeks with substantial overlap of the DHRs between the two timepoints (Figure 3b, left panel). It is apparent from the ChIP-seq signal that the pattern of the non-overlapping DHRs at both 5 and 10 weeks were consistent with a persistence over time although not reaching the 1.5-fold change threshold at both time points. Several examples of specific regions of persistent H3K27ac changes are shown in Figure 3b, right panel.

The comparison of αERKO-DES versus αERKO-veh DHRs revealed DES-induced effects on the epigenome that are not mediated by ERα. There were approximately half as many DHRs (both gain and loss) as were observed in the week 5 WT-DES versus WT-veh comparison, consistent with their relative lack of gene expression changes in response to DES exposure (Figures 1a, 3a). There were also an increased number of DHRs at week 10 compared to week 5 in this group suggesting a general increase in epigenome dynamics of adult compared to pubertal mice. Surprisingly, an overlap of this group of these two time points showed almost no overlap (only 134 gain and 127 loss) suggesting a more transient nature of these DES-induced DHRs that are not dependent on ERα. The additional comparison between αERKO-DES and WT-veh supported this finding (Figure 3a).

Heat maps showing the overlap of DHRs (WT-DES versus WT-veh and αERKO-DES versus αERKO-veh) at 10 weeks confirmed these findings with very few overlapping DHR regions (Figure 3c). It was also apparent from the heat maps that αERKO SVs were refractory to the robust change in H3K27ac signal following DES. An interesting finding was the group of DES-induced changes observed only in αERKO mice and not in WT mice suggesting the presence of ERα prevents the accumulation of DES-induced H3K27ac in these locations. Specific examples of these DHRs also showed the robust pattern changes in H3K27ac among the four groups (Figure 3c). These data taken together confirmed that ERα is required for many DES-altered H3K27ac patterns as well as the persistence of these alterations.

To determine the ERα-dependence of DES induced effects on the SV, we determined DHRs of αERKO-DES to WT-DES (Figure 3a; comparison 5). There were many regions of DHR gain on week 5 [15,586] and week 10 [19,288] suggesting a substantial impact of ERα loss on DES induced changes in H3K27ac pattern. There were 2,947 regions of overlap between the two time points showing ERα-dependent H3K27ac patterns were found in many regions of persistent change. DHR loss in these two groups showed a very similar pattern to the DHR gain. These data taken together show H3K27ac patterns are dynamic over development and that neonatal DES alters these patterns in an ERα-dependent manner.

### Neonatal DES exhibits ERα dependent histone H3K27ac association at the TSS and enhancers near persistently altered DEGs

**Figure 4.**
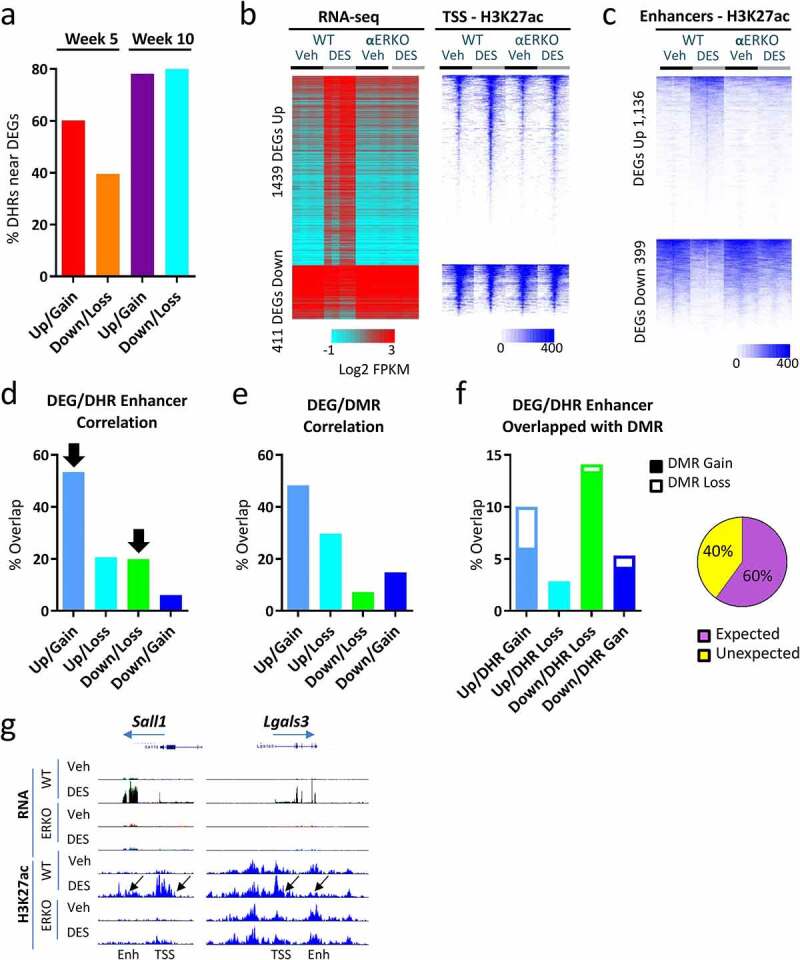
Correlation between DEGs, DHRs and DMRs in SV following neonatal DES exposure. (a) Percent of up- and down-regulated genes (WT-DES versus WT-veh) at week 5 and week 10 that have a DHR gain or loss ±100 Kb of the TSS. (b) Heat map displaying 1,850 persistently altered DEGs (WT-DES versus WT-veh, overlapping week 5 and week 10). RNA-seq (log2 FPKM) and H3K27ac ChIP-seq signal mapping to a ± 5 kb window around TSS in WT and αERKO SVs. Heat maps are split into up- and down-regulated genes. Heat maps are organized by high to low expression in the WT-DES RNA-seq sample and H3K27ac signal is plotted in the same gene order. (c) DHRs ±100 Kb of the TSS of up- or down-regulated genes are plotted. Heat map is organized by high to low signal in the WT-DES sample. (d) Percent overlap of DHR (gain or loss) and DEG (up or down) is plotted. Black arrows indicated the expected correlation of DHR and DEG direction. (e) Percent overlap of DMR (gain or loss) and DEG (up or down) is plotted. (f) Overlap of DEG/DHR categories from Figure 4d with DMRs are plotted in a stacked bar chart with open part of the bar as percentage of DMR gain and solid part of the bar as DMR loss. Pie chart indicated the proportion of expected and unexpected DMR direction. (g) Examples of the RNA-seq and H3K27ac at the TSS and enhancers of persistently altered genes.

To investigate the relationship between the DES-altered transcriptome and H3K27ac modifications in SVs of WT mice, we focused on WT-DES versus WT-veh DEGs. An initial analysis of week 5 and week 10 DEGs and DHRs found ± 100 Kb of the TSS (including the TSS) revealed correlation between the direction of gene expression and the direction of H3K27ac association (Figure 4a).

At 5 weeks, 60% of up-regulated DEGs had increased H3K27ac nearby but only 40% correlation in direction with down-regulated genes. By 10 weeks, the correlation was much higher with ~80% correlation for both up- and down-regulated genes suggesting more influence of this modified histone on nearby genes in adulthood than in puberty (Figure 4a).

The high correlation of gene expression direction and H3K27ac association led us to perform further analysis of the locations of these DHRs (TSS versus enhancers). Because week 10 DEGs were largely overlapped with week 5 DEGs in the same direction with a high degree of correlation of associated H3K27ac at this time point, we restricted further analysis to the 1,850 persistently DES-altered genes (Figure 1d, comparison 2) and used week 10 gene expression and H3K27ac ChIP-seq data. We first generated heatmaps of RNA-seq and corresponding H3K27ac ChIP-seq signal at the TSS of 10-week-old mice split into up- and down-regulated DEGs (Figure 4b, left panel). For the up-regulated genes, there was a substantial increase in gene expression in the WT-DES group compared to the WT-veh group. This increase was completely prevented in the αERKO-DES group. Increased H3K27ac ChIP-seq signal at the TSS of WT-DES was highly correlated with increased gene expression and was also prevented in αERKO mice. In contrast, there was accumulation of H3K27ac at the TSS of down-regulated genes but only slight reductions in WT-DES compared to the other three groups suggesting differences in this mark are not as tightly associated with decreased gene expression (Figure 4b, right panel).

Next, we identified enhancers by restricting DHRs that were within ± 100 kb of the TSS (excluding the TSS ± 5Kb) of the 1,850 persistently DES-altered genes at 10 weeks of age. Of the 1,850 DEGs, 1,535 (83%) had a DHR nearby outside of the TSS. Heat maps of the H3K27ac ChIP-seq signal generally showed increased H3K27ac at enhancers near up-regulated genes and decreased H3K27ac near down-regulated genes (Figure 4b). The lack of ERα prevented this change in H3K27ac association near both up- and down-regulated genes showing that ERα was required for these changes in the epigenome. To determine the impact of ERα on these H3K27ac patterns, we determined if the αERKO-DES sample were refractory to the change (Figure 4c). All DES-altered enhancers except one were dependent on ERα confirming the requirement for ERα on DES-induced epigenome changes. To further investigate the degree of correlation, we split the DEGs and H3K27ac data into four groups: 1) DEGs Up/H3K27ac Gain, 2) DEGs Up/ H3K27ac Loss, 3) DEGs Down/H3K27ac Loss and 4) DEGs Down/H3K27ac Gain. Of the 1,535 DEGs that had a DHR nearby 1,125 (73%) had a DHR in the expected direction, up-regulated DEG with DHR gain or down-regulated DEG with DHR loss, suggesting an influence of H3K27ac occupancy found near these genes (Figure 4d).

DNA methylation status influences H3K27ac binding at enhancer regions [44,45]. To determine if changes in histone H3K27ac were due to DNA methylation changes, we first determined the percentage of the 1,850 persistently altered DEGs that have a DMR gain or loss ±100 Kb of the TSS but excluding the TSS (WT-DES SV versus WT-veh SV at 10 weeks) determined previously [1] (Figure 4e). Of note, ~90% of all DES-induced DMRs were observed in introns and intergenic regions [1]. DMRs were found near 1,375 (74%) of DES altered genes and DMRs were more often found near up-regulated genes [1,073] than down-regulated genes (302). In general, DNA methylation regions should be inversely correlated with gene expression; however, only 44% of the DEGs had the expected DMR direction nearby suggesting less of a role for DNA methylation compared to histone H3K27ac association on nearby gene expression. Recent studies show DNA methylation status can be correlated with gene expression if located in specific regions such as gene bodies [46,47]. To insure the DMR directionality comparison was not due to genomic location, we split the DMRs into the three categories, gene body, intergenic, and promoter regions (figure S2a). When we overlapped the up and down regulated DEGs with the three categories of DMRs based on region, a similar pattern was observed in all three groups suggesting the location of the DMR did not account for influence on nearby gene expression (figure S3a, S3b). Next, we overlapped each of the four sets of correlated DEG/DHR categories with all DMRs observed at 10 weeks of age (WT-DES versus WT-veh) determined previously [1] (Figure 4e). There was very little overlap of DMRs (<15%) in any of the four categories (Figure 4f). DMR regions that were overlapped with DHR gain did not exhibit a clear expected pattern of DMR loss. A summary of the direction of DMR change in relation to the DHR change showed only 60% of the DMR change was in the expected direction (DMR loss/DHR gain). However, a small subset of regions of DHR loss near up- or down-regulated genes had the expected DMR gain in 96% of cases of overlap suggesting a potential cooperation between these two epigenetic mechanisms for these limited specific locations (Figure 4f). These data suggest DNA methylation patterns are largely not responsible for alterations in histone H3K27ac association for most regions. Examples of the RNA-seq and H3K27ac at the TSS and enhancers of persistently altered genes is shown in Figure 4g.

### DES-altered transcriptome, histone H3K27ac, and DNA methylation aberrances appear to be predominantly tissue-specific in mouse reproductive tissues

**Figure 5.**
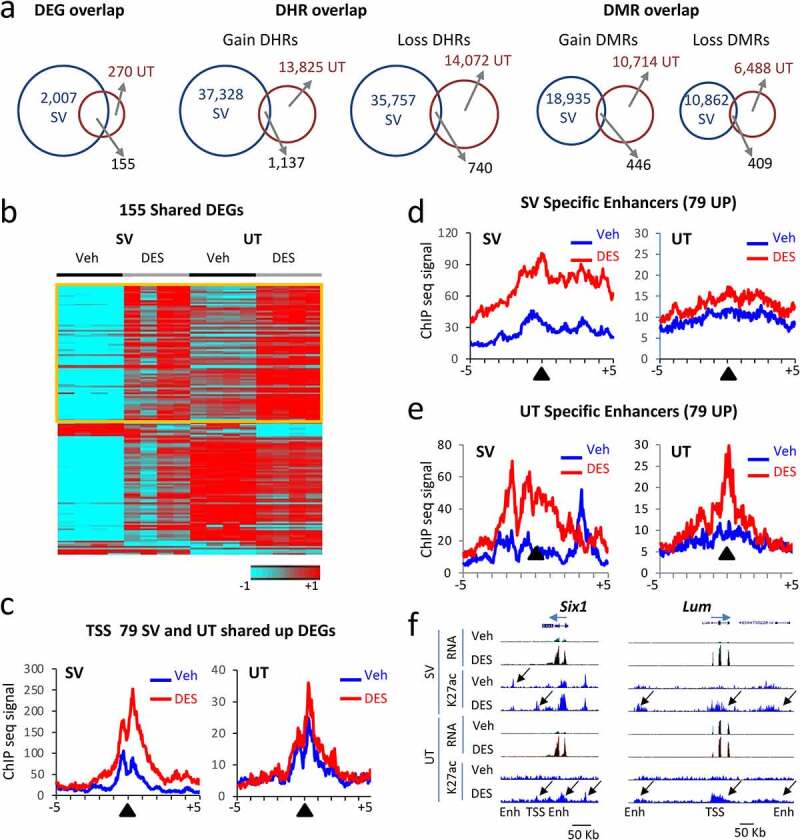
Correlation between mRNA DEGs and H3K27ac DHRs in adult mouse SV and uterus (UT) tissues following neonatal DES exposure. (a) Overlap of DEGs, DHRs, and DMRs between SV and UT at 10 weeks. (b) Heat map displaying 155 DEGs in common between SV and UT at 10 weeks (WT-DES versus WT-veh). Signal plotted is RNA-seq (log2 FPKM) and heat maps are organized by high to low expression in the WT-DES SV sample (c) Metaplots of H3K27ac ChIP-seq signal mapping to a ± 5 kb window around TSS of 79 upregulated genes in WT-veh and WT-DES SV and UT (d) Metaplot of SV specific enhancers, DHRs ±100 Kb of the 79 upregulated genes in SV sample. (e) Metaplot of UT specific enhancers, DHRs ±100 Kb of the 79 upregulated genes in UT sample. (f) UCSC genome browser tracks of example DEGs with DHRs (*Six1* and *Lum*; transcription direction indicated by a blue arrow). TSS and enhancer (Enh) are indicated below the tracks. Sample is indicated on the left and black arrows indicate differential regions of H3K27ac ChIP-seq signal.

To explore whether early DES exposure results in similar or tissue-specific transcriptomic and epigenomic changes in adulthood, we integrated the RNA-, H3K27ac ChIP-, and DNA methylation ChIP-sequencing data between week 10 SV and week 10 ovariectomized uterus (UT) (Figure 5a).

Of note, there were many more DEGs in the SV group compared to the UT group (2,007 versus 270); this is unsurprising because the males were intact with circulating hormones whereas the females in this study lacked sex hormones due to ovariectomy. Of the 270 UT DEGs, 155 (57%) overlapped with SV DEGs suggesting some DES-induced gene expression changes were not tissue specific, but DES sensitive (Figure 5a, left panel). There was a robust DES-induced change in H3K27ac in the UT with 35,757 DHR gain and 14,072 DHR loss. However, overlap of DHRs between SV and UT showed very few in common (3%) suggesting these changes are largely tissue or gene selective (Figure 5a, middle panel). There was also a large number of DES-induced DNA methylation changes with 10,714 DMR gain and 6,488 DMR loss. Again, there was very little overlap between UT and SV (3%) suggesting a large degree of tissue specificity of this chromatin feature (Figure 5a, right panel). To determine if UT had tissue-specific alterations in the epigenome near DEGs, DHRs (figure S4a) and DMRs (figure S4b) data were analysed for comparison of UT WT-DES versus WT-veh. Only 44% of up-regulated genes and 39% of down-regulated genes had the anticipated DHR gain or loss nearby. There was even less correlation with gene expression and the anticipated DMR direction in the UT with 25% of up-regulated genes having a DMR loss and 35% of down-regulated genes having a DMR gain.

To determine if there were specific regions near DEGs that were shared between SV and UT, we first generated a heat map of expression of the 155 overlapping DEGs (Figure 5b). Of these 155 DEGs, 79 upregulated DEGs were increased in both SV and UT (table S4). An analysis of H3K27ac ChIP-seq signal at the TSS of these increased genes showed increases in H3K27ac ChIP-seq signal in WT-DES versus WT-veh for both SV and UT although the signal was much lower in the UT as well as the difference between DES and veh (Figure 5c).

To determine if enhancer regions exhibited tissue-specific association, we first generated metaplots of enhancers derived from SV WT-DES versus SV WT-veh DHRs that were ±100 Kb of the TSS of the 79 genes increased in both SV and UT (Figure 5d). There was increased H3K27ac signal in both SV and UT samples when comparing WT-DES to WT-veh although the difference was more apparent in SV. Next, metaplots were generated using DHRs found when comparing UT WT-DES versus UT WT-veh ±100 Kb of the TSS of the 79 up-regulated DEGs (Figure 5e; figure S3a). These also showed robust increases in H3K27ac in WT-DES versus WT-veh in both SV and UT. The shape of the H3K27ac ChIP-seq signal at enhancers was vastly different between the two tissues with more apparent spreading (wider curve) in the SV samples compared to the UT samples. There was also a peak in the SV WT-veh that was shifted to the right of the enhancer peak suggesting some other type of regulation in these regions in this tissue compared to UT where no peak was observed in UT WT-veh. The sharp peak observed in the UT WT-DES versus WT-veh suggests a tighter regulatory region in the UT compared to the SV in these locations. These data suggest some enhancers near non-tissue specific DEGs have tissue specific corresponding DHRs.

To determine if there are DES specific targets of H3K27ac changes that are not tissue dependent, an overlap of DHRs in SV and UT near the 79 upregulated DEGs revealed 21 genes that have a gain DHR, and 4 genes have a loss DHR in the same location (figure S5). One of these genes is *Six1*, a homeobox transcription factor that is a biomarker for uterine adenocarcinoma [48,49]. Another gene of interest on this list is *Top2a*, a gene highly expressed in stem cell populations and known to play a role in endometrial cancer [50,51]. Also among these genes is *Foxm1*, a gene responsible for epithelial stem/progenitor cell expansion [52]. This pattern of gene expression and correlated DHR at a nearby enhancer is demonstrated by genome browser tracks of *Six1* (Figure 5f). Another gene, *Lum* is among the overlapping 79 upregulated DEGs and although these peaks did not meet the cut-off criteria for a DHR in both tissues, the pattern of overlapping H3K27ac occupancy in both SV and UT is still evident (Figure 5f). These data suggest there is a DES specific epigenetic signature near commonly altered genes that is not tissue dependent.

## Discussion

Our previous studies showed that early exposure to DES impacts mouse SV development in adulthood and DES-altered gene expression aberrances were through ERα [22,23]. Recently, we also reported that DES exposure dynamically changes DNA methylation in SV tissue at adulthood [1,21]. To further evaluate the mechanistic effect of neonatally administered DES in the mouse SV tissue, we examined the transcriptomic and epigenetic alterations during SV development from puberty into adulthood in the current study. A summary of the findings observed herein and in our previous studies is shown in figure S6 [1,15,23]. We showed that i) ERα protected against reduction of SV weight caused by developmental DES exposure. ii) ERα mediated mRNA and lncRNA transcriptome aberrances following DES exposure. iii) DES-altered histone H3K27ac modifications were predominantly ERα dependent. iii) DES-altered DNA methylation changes were partially ERα dependent. v) DES-altered transcriptome aberrances were persistent over time and correlated with histone H3K27ac and DNA methylation status changes but generally not in the same locations. In addition, DES-altered gene expression and epigenome changes are predominantly tissue dependent with differences between SV and UT; however, a small subset of these changes are observed in both SV and UT suggesting some changes are DES dependent and not tissue-specific.

The mechanism underlying persistent changes is male reproductive tract function following neonatal DES exposure is not well understood; therefore, we recently investigated changes in the epigenome in this model. We previously described the substantial impact on DNA methylation in the adult SV in this model that is largely ERα dependent, but studies on other types of epigenetic changes are lacking [1]. The addition of histone H3K27ac alterations following DES exposure presented herein further characterizes the underlying epigenetic changes that occur over time following DES exposure. There are extensive ERα-dependent changes in H3K27ac association following neonatal DES exposure; however, these changes largely do not overlap DNA methylation changes suggesting more than one mode of epigenetic change. This type of comprehensive study will give us insight into potential functional consequences of environmental oestrogen exposures on the epigenome during critical periods of differentiation of these tissues. In future studies, delineation of the epigenetic signature of developmental oestrogen exposure will aid in our understanding of male infertility and potentially lead to ways to improve these phenotypes in the human population.

There were a large number of gene expression changes in the SV at week 5 that were transient in nature and less dependent on ERα than in the adult SV suggesting DES alters pubertal dependent gene expression changes in a non-ERα mediated way. The enrichment of GO categories in this group of DEGs was involved in cellular growth and development supporting this idea. One explanation for these transient pubertal changes may be the negative impact on androgen signalling in the male reproductive tract following neonatal DES exposure [15,19,22,25]. The failure to acquire full androgen signalling during puberty may substantially alter gene expression when compared to normal signalling activity in WT SV. Another explanation could be cellular differentiation is altered following neonatal DES exposure such that gene expression is altered similar to the alteration to *Hox* and *Wnt* genes resulting in posteriorization that occurs in the female reproductive tract following the same treatment schedule [34,53–55]. In fact, *Hox* and *Wnt* genes are altered in the SV following neonatal DES exposure suggesting differentiation is adversely affected [15]. Additional hormone-specific experiments over more time points earlier in development would help elucidate the impact of these two possibilities and would be of interest for future studies.

The persistent DES induced gene expression changes observed in the adult SV were characterized by their role in immunity and inflammatory response. This is consistent with the inflammatory pathologies observed in male reproductive tract tissues following developmental exposures to oestrogenic chemicals [16,56–58]. In addition, male reproductive tract tumours have been described previously following developmental exposure to DES [17,18,59]. Chronic inflammation causes tumours in many tissues and may be an underlying cause of the reproductive tract abnormalities in mice exposed developmentally to oestrogenic chemicals [60]. Further comparisons of the persistent DES DEGs revealed high overlap with perturbations of *Pten* suggesting a potential role for the Pten signalling pathway in the tissue disruption in DES exposed mice. One of the models with very high overlap of gene expression in the same direction was a conditional deletion of *Pten* in prostate epithelium where prostate tumours formed by three months of age [61]. In addition, Pten deletion in this model activated inflammatory responses very early in the progression of tumour formation [62,63]. These data taken together suggest that neonatal DES exposure alters inflammatory pathways, and these changes may contribute to hyperplasia and tumour formation in male reproductive tract tissues.

RNA-seq data from both timepoints revealed transcriptional differences in genes commonly associated with regulating the epigenome such as *Kat, Kmt, Kdm*, and *Hdac* family members. There is a careful, concerted control of gene transcription by modifying histones in response to cellular cues [27]. Disruption of this control on a global scale could lead to aberrant gene expression as has been shown in several model systems [35–39]. One example of this is the repression of the histone methyltransferase, *Smyd3*, using siRNA and the resulting decrease in histone H3K4me3 at the TSS of oestrogen stimulated genes [38]. Another example is the conditional uterine deletion of *Ezh2* (enzyme required for methylation of histone H3K27) where the levels of histone H3K27me3 are reduced and that is accompanied by increases in gene expression and cell division [39]. Gene expression changes observed in the present study were predominantly mediated by ERα. Similar impacts on chromatin modifying enzymes were reported in the uterus following neonatal DES exposure during the time of treatment [34]. These DES-induced global alterations in multiple chromatins modifying enzymes in the SV could impact the normal control of gene expression by affecting chromatin structure and function. As a functional output of altered chromatin modifiers, histone H3K9ac and H3K27me3 were altered following neonatal DES exposure and these changes were also mediated by ERα. These changes were in conflict with some changes in specific chromatin modifying enzymes while in agreement with others suggesting additional complexity in the regulation of modified histones. There was no apparent impact on the global levels of H3K27ac between treatment groups but there were extensive changes in this mark at specific locations. Further research into the impact of DES-induced alterations in chromatin modifying enzymes will be needed to determine how much DES-induced gene expression is controlled by this effect.

The interplay between histone modifications and DNA methylation has only recently been investigated. DNA methylation influences the occupancy of methylated histones, H3K4me3 and H3K4me1 to differentiate between promoters and enhancers [45]. The presence of DNA methylation at the promoter generally negatively impacts transcription and has low H3K4me3 association. Moderate levels of DNA methylation at enhancers are coordinated with H3K4me1 association and indicates a poised or primed enhancer that is not necessarily active. In contrast, H3K27ac occupancy is inversely coordinated with DNA methylation at active enhancers [45]. In addition, forced DNA methylation in targeted regions results in reduced H3K27ac confirming the dependence of H3K27ac on DNA methylation status [44]. Histone H3K27ac occupancy of enhancers near DES-altered genes was highly coordinated with gene expression, increased gene expression with increased H3K27ac or decreased gene expression with decreased H3K27ac. DNA methylation status near these genes was much less coordinated with only 40% of DEGs with the anticipated inverse DMR change. Recent studies show the inverse correlation of DNA methylation and gene expression is not always the case such as when the DMR is located in gene bodies [46,47]. To address this, we split the DMRs into promoters, gene bodies and intergenic regions but there was still very little overlap in the anticipated direction. In addition, the gene expression coordinated DHR occupied enhancers were generally not overlapped with DMRs suggesting DNA methylation changes did not dramatically influence H3K27ac association in these regions. The one exception to this occurred in 15% of DES down-regulated genes with a nearby DHR loss that also had a gain in DNA methylation. These limited locations may exhibit histone H3K27ac loss due to increased DNA methylation. These data suggest most DES-induced gene expression changes are due to ERα-dependent histone H3K27ac association at enhancers that is independent of DNA methylation.

In summary, here we took a global approach to understand how early exposure to an endocrine active chemical such as DES impacts the development of mouse reproductive tract tissues. We found that the SV transcriptome is altered following DES exposure, including mRNA and lncRNA aberrations are predominantly mediated by ERα. Many of the DES-altered genes that are persistent over time are associated with inflammation and immune response which supports the pathology observed in DES exposed male reproductive tract tissues. DES exposure also dynamically changes histone H3K27ac association near altered genes in the anticipated direction and these changes are largely ERα dependent. DNA methylation changes were also observed near DES altered genes, but the anticipated direction of change was not as consistent as histone H3K27ac association suggested less of a role for DNA methylation in influencing gene expression. In addition, DNA methylation changes generally did not overlap with histone H3K27ac changes and therefore lack influence over this mark. Integration of DES induced changes in gene expression and epigenome such as histone H3K27ac in male and female tissues revealed significant tissue specificity. However, a small subset of genes appeared to be tissue independent. These data taken together demonstrate developmental exposure to oestrogens impacts both histone H3K27ac and DNA methylation association largely independently through ERα. However, histone H3K27ac association exhibits a higher coordination with nearby gene expression than DNA methylation. DES induced changes in the epigenome influence nearby gene expression in a persistent manner that disrupts tissue development and function later in life. The accumulating evidence for the consequential developmental exposure and resulting epigenetic effects associated with such tissue disruption leads to an unfortunate conclusion of negative permanent clinical outcomes for affected individuals.

## Supplementary Material

Supplemental Material
