# Supplementary material for: Multiple tissue-specific epigenetic alterations regulate persistent gene expression changes following developmental DES exposure in mouse reproductive tissues
Source: Epigenetics. 2022 Nov 3;18(1):2139986. doi: 10.1080/15592294.2022.2139986 (PMC9980695; doi:10.1080/15592294.2022.2139986)
Supplement: Supplemental Material [file KEPI_A_2139986_SM9214.zip › Supplementary/Jefferson et al_SM final to Epi.docx]

*SUPPLEMENTARY MATERIALS*

## Multiple tissue-specific epigenetic alterations regulate persistent gene expression changes following developmental DES exposure in mouse reproductive tissues

Tanner B. Jefferson, Tianyuan Wang, Wendy N. Jefferson, Yin Li, Katherine J. Hamilton, Paul A.

Wade, Carmen J. Williams, and Kenneth S. Korach

figure S1, Related to Method page 2

figure S2, Related to Figure 3 page 3

figure S3, Related to Figure 4 page 4

figure S4, Related to Figure 5 page 5

figure S5, Related to Figure 5 page 6

figure S6, Related to Discussion page 7

### Birth


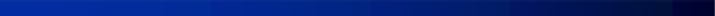

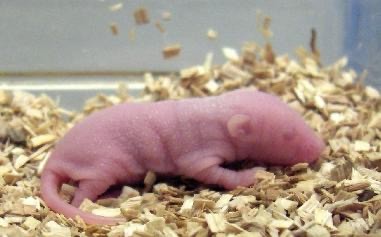

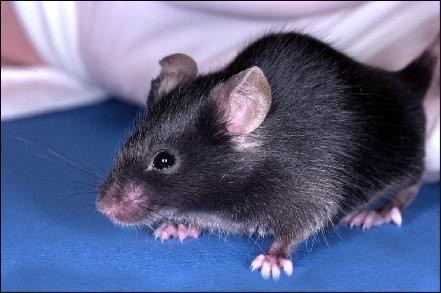


**Puberty Adult**

veh or DES (2 µg/day) day 1-5

**Week 5 Week 10**

**figure S1, Related to Method. Timeline for DES neonatal treatment.** Experimental design of DES injection starting at birth through PND5 and collection of tissue at 5 and 10 weeks.

2

# a


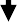

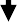

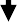


-5

kB

TSS

TES

Enhancers

Upstream 5’-UTR

Exons/Introns

3’-UTR Downstream

## Distribution of DHRs (WT: DES *vs*. veh)

**60 60**

**WT week 5 loss me WT week 10 loss me**

**WT week 5 gain me WT week 10 gain me**

**40 40**

**% of ditribution**

**% of ditribution**

**20 20**

**0 0**

## Distribution of DHRs (αERKO: DES *vs*. veh)

**60 60**

α **ERKO week 5 loss me**

α **ERKO week 10 loss me**

α **ERKO week 5 gain me**

α **ERKO week 10 gain me**

**40 40**

**% of ditribution**

**% of ditribution**

**20 20**

**0 0**

### figure S2, Related to Figure 3. Distribution of DHRs from H3K27ac ChIP-Seq analysis in WT and αERKO mouse SVs during normal development and after neonatal DES exposure.

(a) Genetic map of *Esr1*. (b) Distribution of sites with altered histones in WT mice in either

a vehicle treated SV, or a DES treated one. (c) Distribution of sites with altered histones in αERKO in either a vehicle treated SV, or a DES treated one.

3

# a

| **1. Gene body** |  |  |  |  |
| --- | --- | --- | --- | --- |
| DEGs-category | Up | Up | Down | Down |
| DMR-category | Gain | Loss | Loss | Gain |
| Number of DEGs | 509 | 278 | 66 | 169 |
| **2. Intergenic** |  |  |  |  |
| DEGs-category | Up | Up | Down | Down |
| DMR-category | Gain | Loss | Loss | Gain |
| Number of DEGs | 310 | 227 | 58 | 87 |
| **3. Promoter** |  |  |  |  |
| DEGs-category | Up | Up | Down | Down |
| DMR-category | Gain | Loss | Loss | Gain |
| Number of DEGs | 43 | 23 | 2 | 7 |

b

## DEG/DMR Correlation


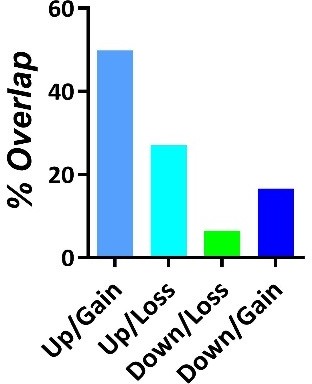

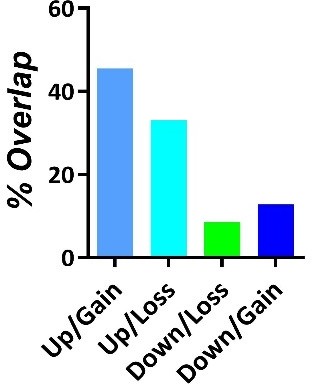

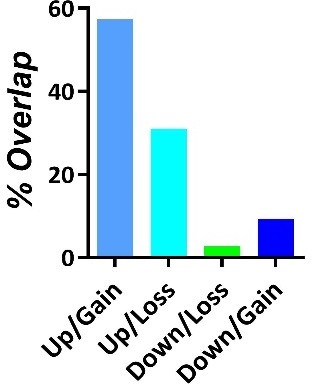
Gene body Intergenic Promoter

**figure S3, Related to Figure 4. The DMR categories and overlap with 1,850 persistent DEGs in WT mouse SVs during normal development and after neonatal DES exposure.** (a) 3 DMR categories. (b) DEG/DMR overlap split into 3 DMR categories from panel A.

4

### Overlap: DES-altered DEGs and DHRs with +/- 100 kb at TSS in week 10 WT Uts


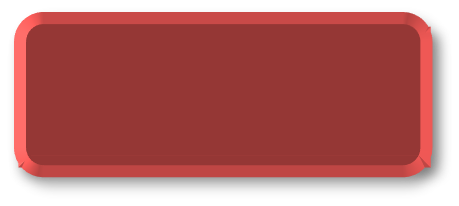

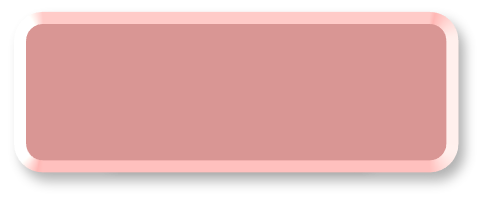

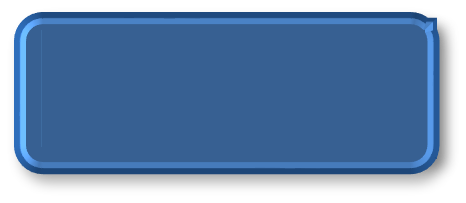

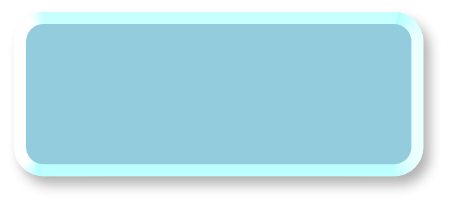

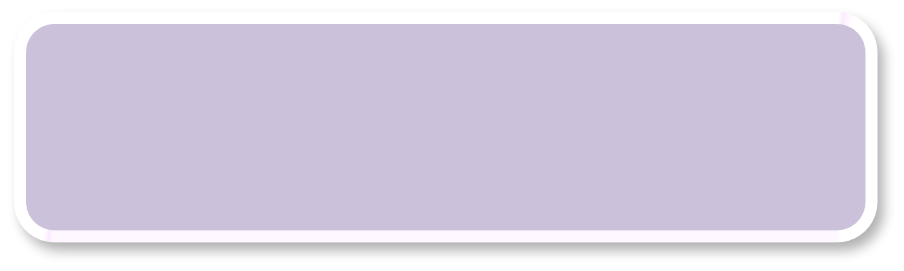

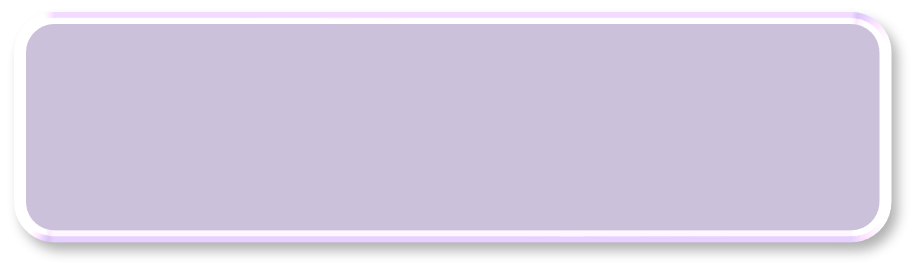


**182 DES-induced genes**

**14,962 gain DHRs**

**243 DES-repressed genes**

**14,812 loss DHRs**

**80 DES-induced genes with 159 gain DHRs (44% of DES-induced genes)**

**95 DES-repressed genes with 138 loss DHRs (39% of DES-repressed genes)**

1. **Overlap: DES-altered DEGs and DMRs with +/- 100 kb at TSS in week 10 WT Uts**


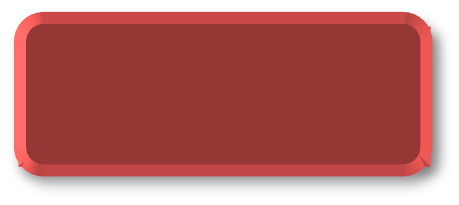

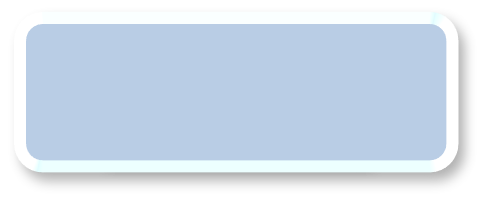

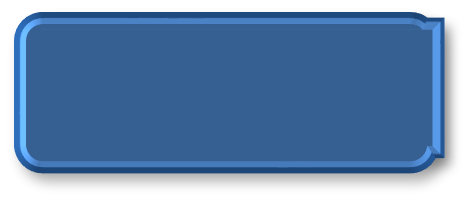

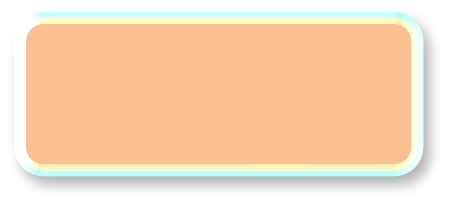

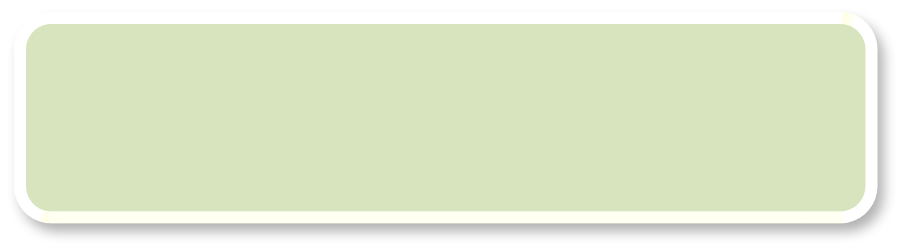

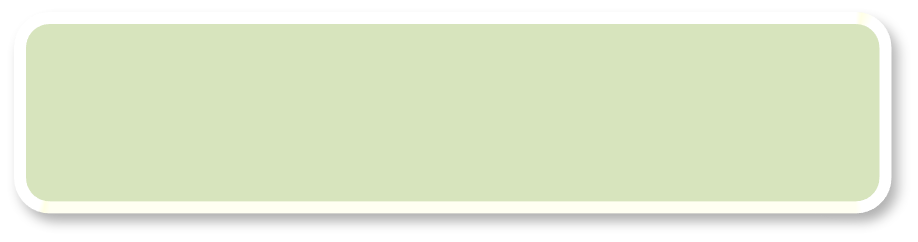


**182 DES-induced genes**

**6,897 loss DMRs**

**243 DES-repressed genes**

**11,160 gain DMRs**

**45 induced genes with 65 loss DMRs (25% of DES-induced genes)**

**84 repressed genes with 148 gain DMRs (35% of DES-repressed genes)**

### figure S4, Related to Figure 5. DES-altered DEGs and DHRs (a) or DMRs (b) integration in adult WT mouse uterine tissues.

| **Gene #** | **Genes with gain DHRs** | | | **Log2 (SV-DES/SV-Veh)** | **Log2(Ut-DES/Ut-Veh)** |
| --- | --- | --- | --- | --- | --- |
| 1 | *Adam12* | | | 3.22 | 0.63 |
| 2 | *Col1a1* | | | 1.73 | 0.80 |
| 3 | *Ctgf* | | | 1.14 | 0.59 |
| 4 | *Cxcl14* | | | 1.78 | 0.52 |
| 5 | *D17H6S56E-5* | | | 3.01 | 1.36 |
| 6 | *Eln* | | | 1.92 | 0.63 |
| 7 | *Gpr133* | | | 2.09 | 0.77 |
| 8 | *Iqgap3* | | | 1.27 | 0.86 |
| 9 | *Itgb2* | | | 3.67 | 0.84 |
| 10 | *Kif22* | | | 1.72 | 0.99 |
| 11 | *Lbh* | | | 2.38 | 0.65 |
| 12 | *Lgi2* | | | 3.96 | 1.05 |
| 13 | *Mfap2* | | | 2.80 | 1.17 |
| 14 | *Mfap4* | | | 1.45 | 0.78 |
| 15 | *Mgp* | | | 2.41 | 1.12 |
| 16 | *Msln* | | | 5.01 | 1.24 |
| 17 | *Six1* | | | 2.14 | 3.60 |
| 18 | *Sparc* | | | 1.12 | 0.61 |
| 19 | *Sulf1* | | | 1.52 | 0.54 |
| 20 | *Thbs2* | | | 1.76 | 0.54 |
| 21 | *Top2a* | | | 1.26 | 0.88 |
| **Gene #** | **Genes with loss DHRs** | | | **Log2 (SV-DES/SV-Veh)** | **Log2 (Ut-DES/Ut-Veh)** |
| 1 | *Ctgf* | | | 1.14 | 0.59 |
| 2 |  | *Foxm1* | | 1.30 | 0.80 |
| 3 |  | *Igj* |  | 7.34 | 0.78 |
| 4 | *Slpi* | | | 5.87 | 2.37 |

**figure S5, Related to Figure 5. The gene lists of DES-altered DEGs with gain DHRs or DEGs with loss DHRs in both adult mouse SV and uterine tissues.**

**Birth**


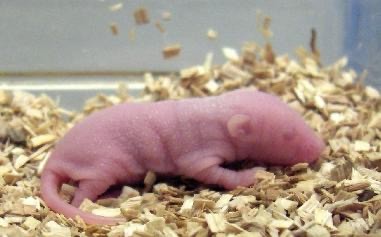

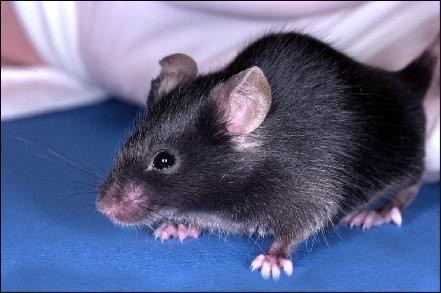


**Puberty**

**Adult**

**DES exposure (day 1-5)**

### Week 3

**Week 5 Week 10**

**i.** ERα protects reduction of SV weight by DES developmentally.

2.5

2.0

**Week 3**

*******

SV weight (mg)

1.5

1.0

0.5

0.0 WT

WT αERKO αERKO

40

30

**Week 5**

*******

SV weight (mg)

20

10

0 WT


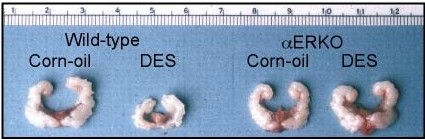
WT αERKO αERKO

150

100

**Week 10**

*******

SV weight (mg)

50

0 WT

WT αERKO αERKO

-veh

-DES

-veh

-DES

-veh

-DES

-veh

-DES

### Overlap

-veh

-DES

-veh

-DES

**ii.**

**iii.**

**iv.**

**v.**

DES-altered H3K27ac modification is partially ERα dependent.

DES-altered DNA methylation change is partially ERα dependent.

ERα mediates mRNA transcriptome aberrances following DES exposure.

ERα mediates long non- coding RNA transcriptome aberrances following DES exposure.

DES-altered mRNA gene expression aberrances correlate with histone H3K27ac and DNA methylation status changes.

### Week 5

40,679 gain and loss DHRs (90.9% ERα dependent)

### (% of week 10)

27% DHR

74,962 gain and loss DHRs (78.9% ERα dependent)

overlap

7,219 DEGs

( 95.9% ERα dependent)

86% DEG

overlap

30,653 gain and loss DMRs (97.1% ERα dependent)

12,178 gain and loss DMRs (94.5% ERα dependent)

2% DMR

overlap

### Week 10

2,162 DEGs

(99.6 % ERα dependent)

67% DE

lncRNA overlap

607 DE lncRNAs

(98.7% ERα dependent)

1,188 DE lncRNAs

(90.3% ERα dependent)

**figure S6, Related to Discussion. Summary of that early DES exposure alters epigenetic and transcriptome aberrances in mouse SVs.**

4,077 up

genes

60% with H3K27ac

25% with DNAme

3,142 down genes

73% with H3K27ac

13% with DNAme

1,678 up

genes

73% with H3K27ac

50% with DNAme

484 down genes

85% with H3K27ac

75% with DNAme
